# Diagnostic Value of Breast Lesions Between Deep Learning-Based Computer-Aided Diagnosis System and Experienced Radiologists: Comparison the Performance Between Symptomatic and Asymptomatic Patients

**DOI:** 10.3389/fonc.2020.01070

**Published:** 2020-07-07

**Authors:** Mengsu Xiao, Chenyang Zhao, Jianchu Li, Jing Zhang, He Liu, Ming Wang, Yunshu Ouyang, Yixiu Zhang, Yuxin Jiang, Qingli Zhu

**Affiliations:** Department of Ultrasound, Chinese Academy of Medical Sciences and Peking Union Medical College Hospital, Beijing, China

**Keywords:** computer-aided diagnosis, deep learning, breast, ultrasound, symptomatic

## Abstract

**Purpose:** The purpose of this study was to compare the diagnostic performance of breast lesions between deep learning-based computer-aided diagnosis (deep learning-based CAD) system and experienced radiologists and to compare the performance between symptomatic and asymptomatic patients.

**Methods:** From January to December 2018, a total of 451 breast lesions in 389 consecutive patients were examined (mean age 46.86 ± 13.03 years, range 19–84 years) by both ultrasound and deep learning-based CAD system, all of which were biopsied, and the pathological results were obtained. The lesions were diagnosed by two experienced radiologists according to the fifth edition Breast Imaging Reporting and Data System (BI-RADS). The final deep learning-based CAD assessments were dichotomized as possibly benign or possibly malignant. The diagnostic performances of the radiologists and deep learning-based CAD were calculated and compared for asymptomatic patients and symptomatic patients.

**Results:** There were 206 asymptomatic screening patients with 235 lesions (mean age 45.06 ± 10.90 years, range 21–73 years) and 183 symptomatic patients with 216 lesions (mean age 50.03 ± 14.97 years, range 19–84 years). The sensitivity, specificity, positive predictive value (PPV), negative predictive value (NPV), accuracy and area under the receiver operating characteristic curve (AUC) of the deep learning-based CAD in asymptomatic patients were 93.8, 83.9, 75.0, 96.3, 87.2, and 0.89%, respectively. In asymptomatic patients, the specificity (83.9 vs. 66.5%, *p* < 0.001), PPV (75.0 vs. 59.4%, *p* = 0.013), accuracy (87.2 vs. 76.2%, *p* = 0.002) and AUC (0.89 to 0.81, *p* = 0.0013) of CAD were all significantly higher than those of the experienced radiologists. The sensitivity (93.8 vs. 80.0%), specificity (83.9 vs. 61.8%,), accuracy (87.2 vs. 73.6%) and AUC (0.89 vs. 0.71) of CAD were all higher for asymptomatic patients than for symptomatic patients. If the BI-RADS 4a lesions diagnosed by the radiologists in asymptomatic patients were downgraded to BI-RADS 3 according to the CAD, then 54.8% (23/42) of the lesions would avoid biopsy without missing the malignancy.

**Conclusion:** The deep learning-based CAD system had better performance in asymptomatic patients than in symptomatic patients and could be a promising complementary tool to ultrasound for increasing diagnostic specificity and avoiding unnecessary biopsies in asymptomatic screening patients.

## Introduction

Breast cancer is a leading cause of cancer-related mortality in women worldwide (1). As an important supplementary modality for mammography, ultrasound plays an important role in dense breast tissue. Ultrasound is more suitable for Asian women, most of whom have thinner and denser breast glands and a younger age of onset for breast cancer, than Western women. A multicenter randomized trial across China compared ultrasound and mammography for breast cancer screening in high-risk Chinese women and showed that ultrasound had a significantly higher sensitivity and accuracy than mammography (2). Currently, ultrasound is widely used as the primary screening modality for breast cancer in China (3). However, ultrasounds often lead to a certain number of false-positive lesions and unnecessary biopsies or surgeries because ultrasound has low specificity and positive predictive value (PPV) (4–6). This has become an urgent problem of ultrasound in breast cancer screening in China.

In recent years, a deep learning-based computer-aided diagnosis (CAD) system for breast ultrasound (S-Detect^TM^ for Breast in RS80A; Samsung Medison Co., Ltd., Seoul, Korea) has become commercially available (7). This system has good performance in diagnosing benign and malignant breast lesions and especially in improving the specificity of ultrasound (8). Our early study showed that the deep learning-based CAD had the same diagnostic accuracy as experienced radiologists, and the specificity of the CAD was higher than that of the radiologists, which helped to reduce the number of unnecessary biopsies (9). Our recent study also showed that the deep learning-based CAD had a better performance in the breast benign lesions than the radiologists, especially in fibroadenomas and adenosis (10).

Radiologists often consider clinical factors (such as age, high-risk factors, clinical symptoms, and surgical history) as well as the images to make comprehensive judgments; in contrast, the CAD only considers ultrasound images without any clinical factors. Thus, we believe that the deep learning-based CAD is better at diagnosing asymptomatic patients than symptomatic patients since it only analyzes imaging data. Currently, the major mode of achieving early detection for breast cancer in China is hospital-based opportunistic screening among asymptomatic self-referred women (3), so we proposed CAD may be more helpful in breast cancer asymptomatic screening. To the best of our knowledge, no reports have been published on this topic yet. This study prospectively analyzed the value of deep learning-based CAD in asymptomatic screening patients by comparing with symptomatic patients.

## Materials and Methods

### Patients

From January to December 2018, a total of 409 consecutive patients were examined at the Peking Union Medical College Hospital. All lesions underwent biopsy, and the pathologies were obtained. This prospective study was approved by the institutional review board. Informed consent was obtained from all patients included in the study.

Inclusion criteria were listed as follows:

(1) Had breast lesions clearly visualized by ultrasound;
(2) Underwent biopsy of the lesions and had pathological results;
(3) Provided informed consent.

Exclusion criteria were listed as follows:

(1) Patients who were pregnant or lactating;
(2) Patients who had breast biopsy or were undergoing neoadjuvant chemotherapy or radiotherapy.

**Figure 1:**
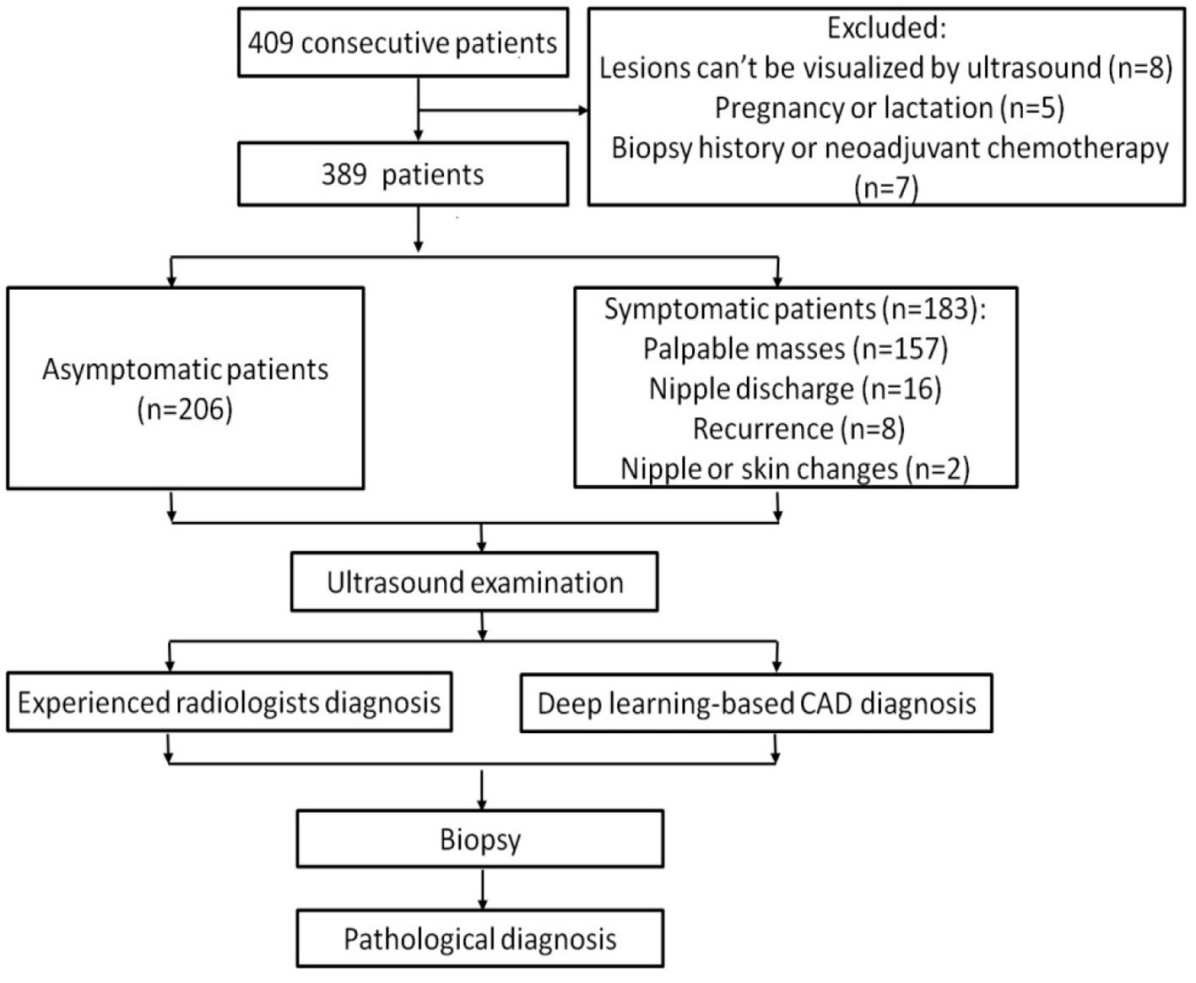
The flow diagram of the study.

Among these patients, 8 women whose lesions can't be visualized by ultrasound, 5 women who were pregnant or lactating and 7 women who had breast biopsy or were undergoing neoadjuvant chemotherapy were excluded. Ultimately, a total of 451 breast lesions in 389 patients were included in this study. The patients were divided into symptomatic and asymptomatic groups. Patients with any clinical manifestations of the breast are classified as symptomatic group, including palpable breast masses, localized pain, nipple discharge, trauma, redness and swelling of the breast, skin changes, nipple retraction, and nipple eczematoid changes. The patients in the asymptomatic group had no symptoms in their breasts and had undergone ultrasound for breast cancer screening. Figure 1 shows the flow chart of study.

### Ultrasound Examination

The ultrasound examinations were performed using a 3–12 MHz linear transducer (RS80A with Prestige, Samsung Medison, Co. Ltd., Seoul, Korea). Two radiologists (QL Zhu and MS Xiao) with 17 and 12 years of experience in breast imaging bilaterally examined the whole breasts of all patients by using ultrasound. The radiologists were aware of the clinical information (history, symptoms, etc.), mammographic results, magnetic resonance imaging (MRI) results, and previous ultrasound results before performing the ultrasound examination. When a breast lesion was detected, two images of the longitudinal and transverse sections of the largest lesion diameter were routinely obtained, and still images were recorded. The lesions were diagnosed by the experienced radiologists based on fifth edition Breast Imaging Reporting and Data System (BI-RADS) by the American College of Radiology (11). The radiologists were blinded to the CAD results when they made the diagnosis for breast lesions. The final diagnosis was classified as follows: category 3, probably benign; category 4a, low suspicion for malignancy; category 4b, intermediate suspicion for malignancy; category 4c, moderate concern for malignancy; and category 5, highly suggesting malignancy. The radiologists were blinded to the pathologic results. The diagnostic cutoff was category 4a. Category 3 lesions were considered benign, while category 4a, 4b, 4c, and 5 lesions were considered malignant.

### Deep Learning-Based CAD Examination

The CAD examination was performed by using deep learning-based CAD software (Samsung Healthcare, South Korea) by the same two radiologists who performed ultrasound examination. The CAD system utilizes large data sets collected from numerous breast exam cases and provides the characteristics of displayed lesion. The CAD applies a novel feature extraction technique and support vector machine classifier. By adopting a deep learning algorithm in the processes of lesion segmentation, analysis of characteristics and assessment, the CAD gives a dichotomized diagnosis whether a selected lesion is benign or malignant according to the proposed feature combinations integrated according to the BI-RADS.

**Figure 2:**
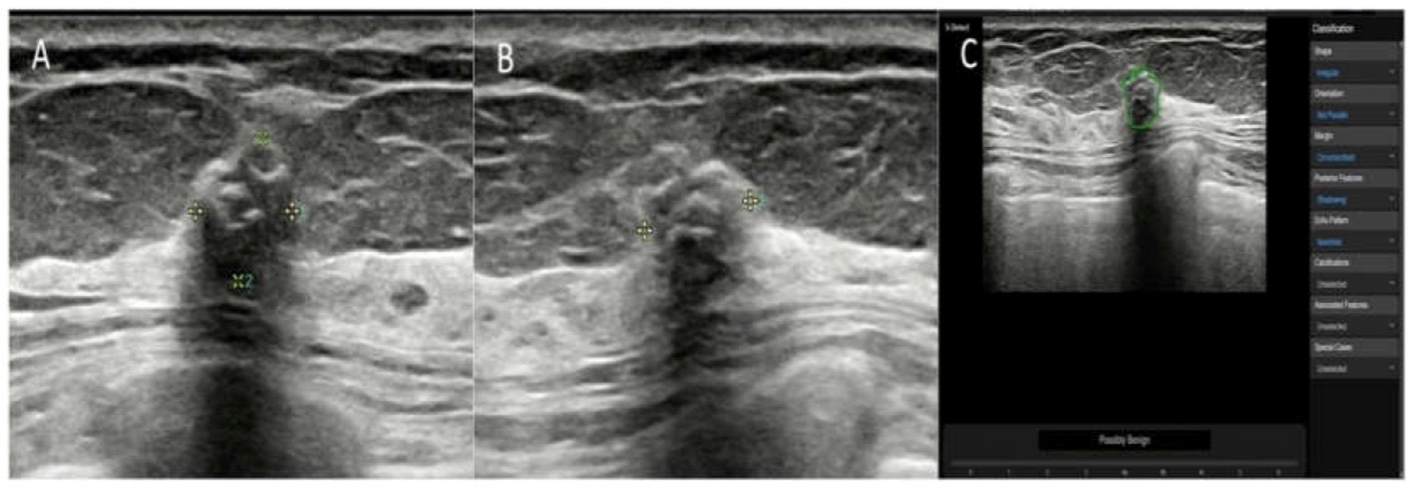
The breast mass of an asymptomatic 57-year-old woman. **(A,B)** The longitudinal section and cross-section of the lesion showed a 6-mm mass with calcifications and posterior shadowing. The orientation is not parallel. The experienced radiologists diagnosed the lesion as BI-RADS 4a. **(C)** A ROI was automatically drawn along the margin of the mass (green line). The raw imaging data were automatically analyzed, and the final diagnosis of the deep learning-based CAD system was a possibly benign tumor. The mass was pathologically proven to be a fibroadenoma.

**Figure 3:**
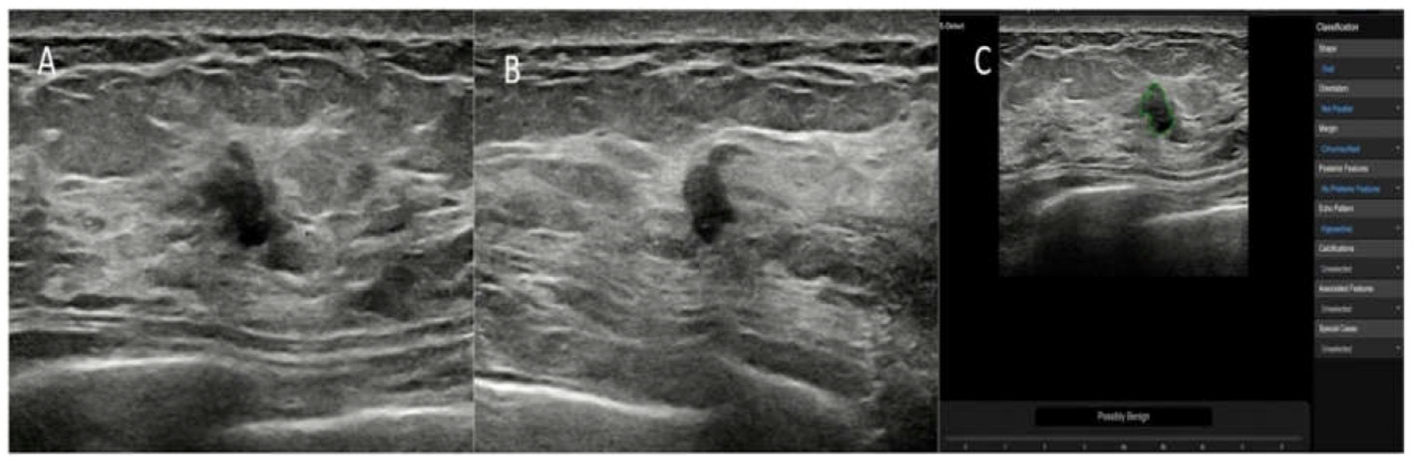
The breast lesion of an asymptomatic 46-year-old woman. **(A,B)** The longitudinal section and cross-section of the lesion showed a 7-mm lesion with irregular shape and ill-defined margins. The diagnosis of the experienced radiologists was a BI-RADS 4b lesion. **(C)** The diagnosis of the deep learning-based CAD system was a possibly benign tumor. The pathological result was fat necrosis.

**Figure 4:**
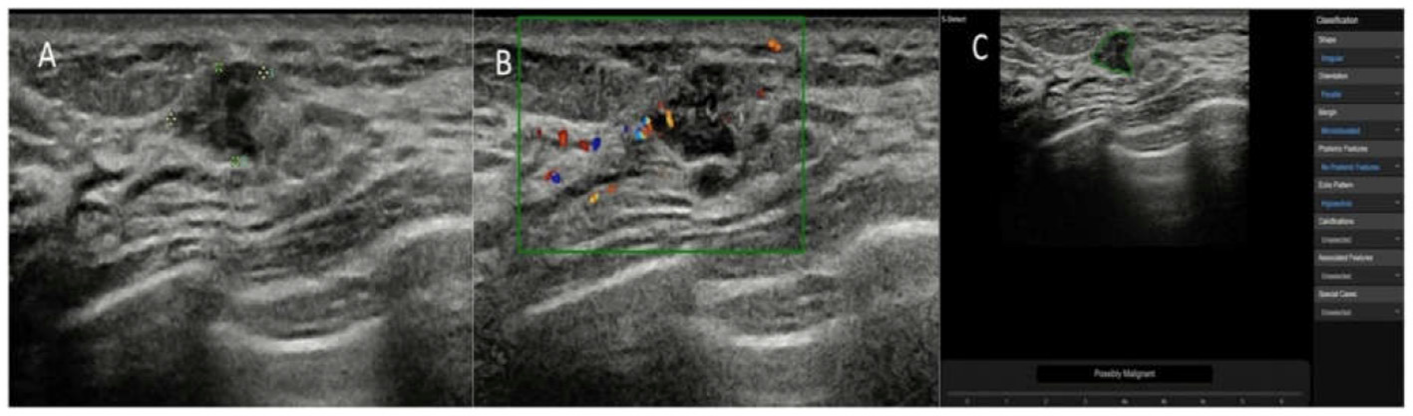
The breast lesion of an asymptomatic 50-year-old woman. **(A)** The longitudinal section of a 9-mm lesion. **(B)** Color Doppler flow imaging of the lesion. The diagnosis of the experienced radiologists was a BI-RADS 3 lesion. **(C)** The diagnosis of the deep learning-based CAD system was a possibly malignant tumor. The pathological result was invasive ductal carcinoma.

On the maximum diameter section of the lesion, the radiologists started the CAD in the center of the lesion. If the maximum diameter of tumor was larger than the machine screen, we selected the most representative section (showing the most suspicious features) of the lesion for CAD to analyze. A region of interest (ROI) was automatically drawn along the border of the lesion. If the automatic outline of ROI was not considered accurate, the radiologists could manually modify the tumor boundary. Based on the given ROI, all of the data and information about the lesion were extracted and analyzed. The CAD system comprehensively analyzed the extracted information, provided a BI-RADS lexicon of the lesions including shape, orientation, margins, pattern and posterior acoustic features, and made a dichotomized diagnosis (possibly benign and possibly malignant) (Figures 2–4). The entire deep learning-based CAD process took only a few seconds.

### Pathological Diagnosis

All of the breast lesions in our study underwent biopsy, and histopathological results were considered the gold standard, including all of the category 3 lesions. The category 3 lesions underwent biopsy according to the patients' choices or patients with high risk factors, including family history and nipple discharge. Immunohistochemical examinations were performed when needed.

### Statistical Analysis

Statistical analysis was performed using SPSS 21.0 (SAS Inc., Cary, NC, USA). The diagnostic performances of the physician and deep learning-based CAD system were analyzed and compared in terms of the sensitivity, specificity, positive likelihood ratio (PLR), negative likelihood ratio (NLR), PPV, negative predictive value (NPV) and accuracy. The 2 × 2 contingency table, chi-square test and McNemar test were used to compare the differences in performance. Receiver operating characteristic (ROC) curves were drawn, and the areas under the ROC curves (AUCs) were calculated. A *p* < 0.05 was regarded as statistically significant.

## Results

In total, 206 asymptomatic screening patients had 235 lesions; the mean age was 45.06 ± 10.90 years (range 21–73 years), and the mean lesion size was 1.44 ± 0.60 cm (range 0.4–4.9 cm). A total of 183 symptomatic patients had 216 lesions, including 16 patients with nipple discharge, 157 with palpable masses, 8 with recurrence after malignant tumor resection, and 2 with nipple depressions or skin changes. The mean age was 50.03 ± 14.97 years (range 19–84 years), and the mean lesion size was 2.42 ± 1.26 cm (range 0.3–9.2 cm). The symptomatic patients were significantly older than the asymptomatic patients (*p* < 0.001). The lesions in the symptomatic patients were significantly larger than those in the asymptomatic patients (*p* < 0.001). The pathological results of the lesions are listed in Tables 1, 2.

**Table 1:** The pathological types of the 220 malignant lesions.

**Malignant lesions**	**Number of asymptomatic lesions (ratio%)**	**Number of symptomatic lesions (ratio%)**	**Total number (ratio%)**
Intraductal carcinoma	13 (16.25)	17 (12.14)	30 (13.64)
Invasive ductal carcinoma, not otherwise specified	56 (70)	98 (70)	154 (70)
Invasive lobular carcinoma	5 (6.25)	5 (3.57)	10 (4.55)
Apocrine carcinoma	1 (1.25)	1 (0.71)	2 (0.91)
Papillary carcinoma	0 (0)	7 (5)	7 (3.18)
Mucinous carcinoma	1 (1.25)	4 (2.86)	5 (2.23)
Neuroendocrine carcinoma	2 (2.5)	0 (0)	2 (0.91)
Malignant phyllodestumours	0 (0)	5 (3.56)	5 (2.23)
Metaplastic carcinoma	0 (0)	1 (0.71)	1 (0.45)
Medullary carcinoma	1 (1.25)	0 (0)	1 (0.45)
Tubular carcinoma	1 (1.25)	0 (0)	1 (0.45)
myofibroblastoma	0 (0)	1 (0.71)	1 (0.45)
Diffuse large B-cell lymphoma	0 (0)	1 (0.71)	1 (0.45)
Total	80	140	220

**Table 2:** The pathological types of the 231 benign lesions.

**Benign lesions**	**Number of asymptomatic lesions (ratio%)**	**Number of symptomatic lesions (ratio%)**	**Total number (ratio%)**
Fibroadenoma	92 (59.35)	34 (44.74)	126 (54.55)
Adenosis	39 (25.16)	15 (19.74)	54 (23.38)
Intraductal papilloma	13 (8.39)	16 (21.05)	29 (12.55)
Phyllodestumour	1 (0.65)	2 (2.63)	3 (1.30)
Chronic inflammation	7 (4.52)	4 (5.26)	11 (4.76)
Granular inflammation	1 (0.65)	3 (3.95)	4 (1.73)
Hamartoma	0 (0)	1 (1.32)	1 (0.43)
Epidermoid cyst	0 (0)	1 (1.32)	1 (0.43)
Cyst	1 (0.65)	0 (0)	1 (0.43)
Fat necrosis	1 (0.65)	0 (0)	1 (0.43)
Total	155	76	231

**Figure 5:**
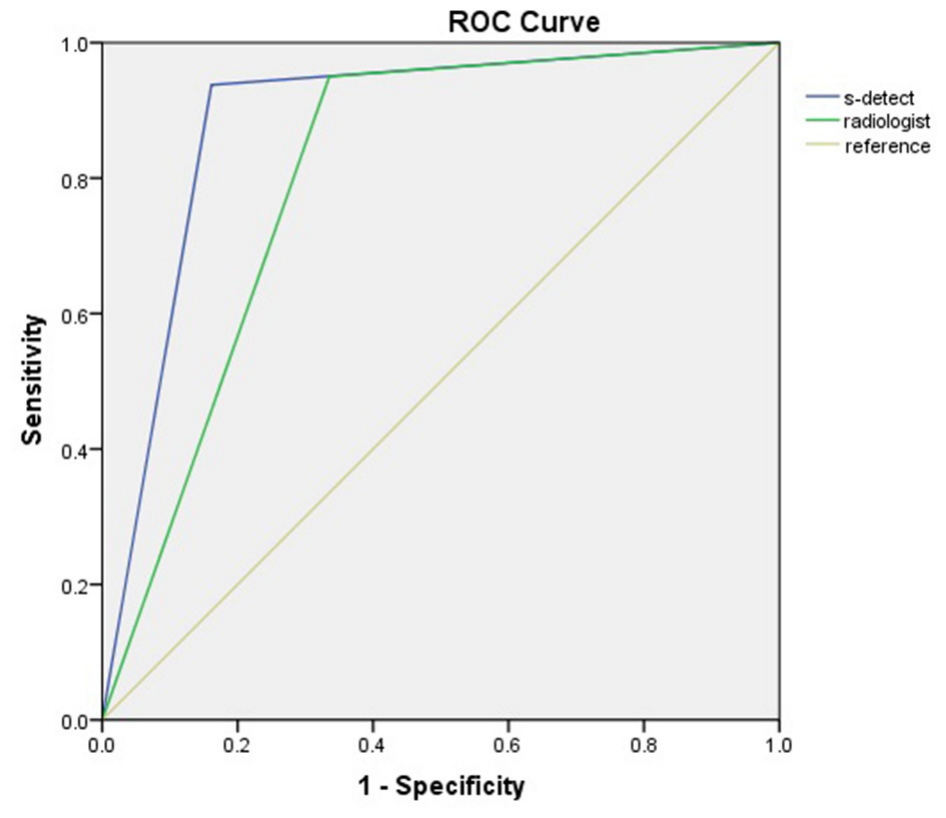
ROC curves of asymptomatic patients.

**Figure 6:**
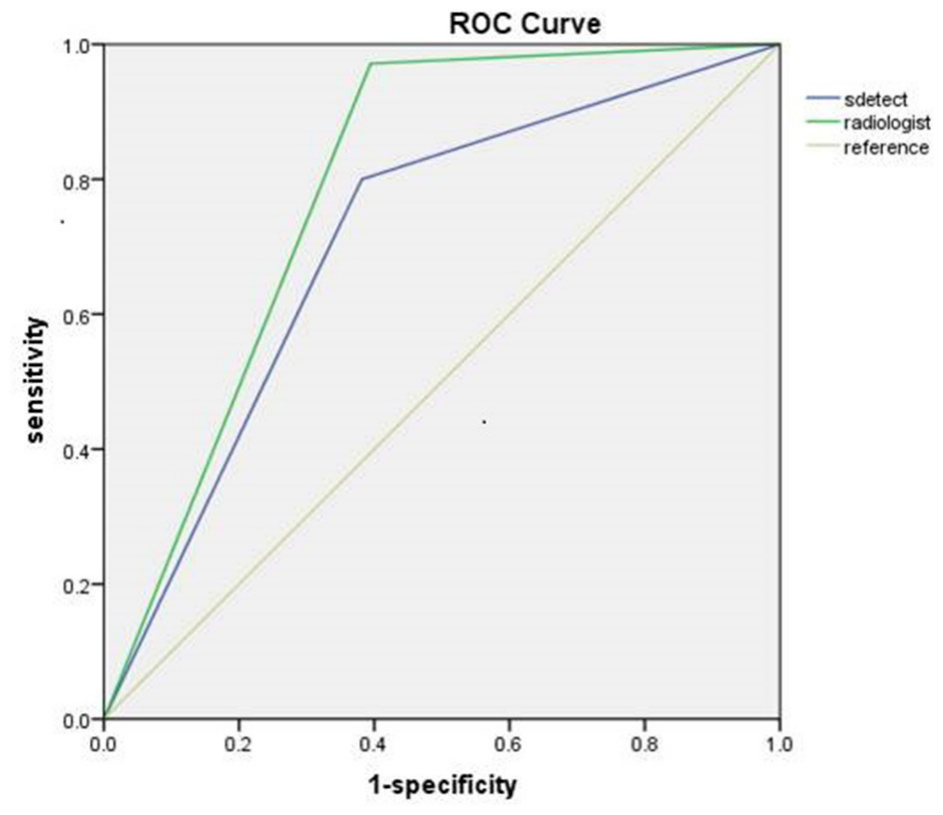
ROC curves of symptomatic patients.

The diagnostic performances of the deep learning-based CAD system and radiologists (asymptomatic patients and symptomatic patients) are shown in Table 3. The diagnostic performances of the deep learning-based CAD system and radiologists for lesions <1 cm (asymptomatic patients and symptomatic patients) are shown in Table 4. The false-positive and false-negative results of the deep learning-based CAD system are shown in Tables 5, 6. The subcategorization of asymptomatic and symptomatic breast lesions by the experienced radiologists is shown in Table 7. The ROC curves are shown in Figures 5, 6.

**Table 3:** The diagnostic performances of the deep learning-based CAD system and experienced radiologists for asymptomatic lesions and symptomatic lesions.

		**SE(%)**	**SP(%)**	**PLR**	**NLR**	**PPV(%)**	**NPV(%)**	**Accuracy (%)**	**AUC**
		**(95%CI)**	**(95%CI)**	**(95%CI)**	**(95%CI)**	**(95%CI)**	**(95%CI)**	**(95%CI)**	**(95%CI)**
Asymptomatic lesions	CAD	93.75	83.87	5.81	0.07	75.00	96.30	87.23	0.89
		(86.01–97.94)	(77.12–89.28)	(4.04–8.36)	(0.03–0.17)	(65.34–83.12)	(91.57–98.79)	(82.28–91.22)	(0.84–0.93)
	radiologists	95.00	66.45	2.83	0.08	59.38	96.26	76.17	0.81
		(87.69–98.62)	(58.43–73.83)	(2.26–3.55)	(0.03–0.20)	(50.34–67.96)	(90.70–98.97)	(70.20–81.47)	(0.75–0.86)
Symptomatic lesions	CAD	80.00	61.84	2.10	0.32	79.43	62.67	73.61	0.71
		(72.41–86.28)	(49.98–72.75)	(1.56–2.82)	(0.22–0.47)	(71.82–85.77)	(50.73–73.57)	(67.20–79.36)	(0.64–0.77)
	radiologists	97.14	60.53	2.46	0.05	81.93	92.00	84.26	0.79
		(92.85–99.22)	(48.65–71.56)	(1.86–3.26)	(0.02–0.13)	(75.22–87.46)	(80.77–97.78)	(78.70–88.85)	(0.73–0.84)

*SE, sensitivity; SP, specificity; PLR, positive likelihood ratio; NLR, negative likelihood ratio; PPV, positive predictive value; NPV, negative predictive value; AUC, area under the receiver operator characteristics curve; 95% CI, 95% confidence interval*.

**Table 4:** The diagnostic performances of the deep learning-based CAD system and experienced radiologists for lesions <1 cm.

		**SE(%)**	**SP(%)**	**PLR**	**NLR**	**PPV(%)**	**NPV(%)**	**Accuracy (%)**	**AUC**
		**(95%CI)**	**(95%CI)**	**(95%CI)**	**(95%CI)**	**(95%CI)**	**(95%CI)**	**(95%CI)**	**(95%CI)**
Asymptomatic lesions	CAD	100.00	88.64	8.80	0.00	77.27	100.00	91.80	0.94
		(80.49–100.00)	(75.44–96.21)	(3.86–20.09)		(54.63–92.18)	(90.97–100.00)	(81.90–97.28)	(0.85–0.99)
	Radiologists	100.00	65.91	2.93	0.00	53.13	100.00	75.41	0.83
		(80.49–100.00)	(50.08–79.51)	(1.95–4.42)		(34.74–70.91)	(88.06–100.00)	(62.71–85.54)	(0.71–0.91)
Symptomatic lesions	CAD	60.00	72.73	2.20	0.55	75.00	57.14	65.38	0.66
		(32.29–83.66)	(39.03–93.98)	(0.77–6.29)	(0.27–1.13)	(42.81–94.51)	(28.86–82.34)	(44.33–82.79)	(0.45–0.84)
	Radiologists	86.67	63.64	2.38	0.21	76.47	77.78	76.92	0.75
		(59.54–98.34)	(30.79–89.07)	(1.06–5.34)	(0.05–0.82)	(50.10–93.19)	(39.99–97.19)	(56.35–91.03)	(0.54–0.90)

*SE, sensitivity; SP, specificity; PLR, positive likelihood ratio; NLR, negative likelihood ratio; PPV, positive predictive value; NPV, negative predictive value; AUC, area under the receiver operator characteristics curve; 95% CI, 95% confidence interval*.

**Table 5:** False positive cases of deep learning-based CAD system.

**False positive cases**	**Number of asymptomatic lesions (ratio%)**	**Number of symptomatic lesions (ratio%)**	**Total number (ratio%)**
Fibroadenoma	6 (24)	8 (27.59)	14 (25.93)
Adenosis	9 (36)	7 (24.14)	16 (29.63)
Intraductal papilloma	6 (24)	8 (27.59)	14 (25.93)
Benign phyllodestumour	0 (0)	2 (6.90)	2 (3.70)
inflammation	3 (12)	4 (13.79)	7 (12.96)
Cyst	1 (4)	0 (0)	1 (1.85)
Total	25	29	54

**Table 6:** False negative cases of deep learning-based CAD system.

**False negative cases**	**Number of asymptomatic lesions (ratio%)**	**Number of symptomatic lesions (ratio%)**	**Total Number (ratio%)**
Intraductal carcinoma	1 (20)	6 (21.42)	7 (21.21)
Invasive ductal carcinoma, not otherwise specified	2 (40)	10 (35.71)	12 (36.36)
Invasive lobular carcinoma	1 (20)	0 (0)	1 (3.03)
Papillary carcinoma	0 (0)	5 (17.86)	5 (15.15)
Mucinous carcinoma	1 (20)	3 (10.71)	4 (12.12)
Malignant phyllodestumours	0 (0)	4 (14.29)	4 (12.12)
Total	5	28	33

**Table 7:** The subcategorization of asymptomatic and symptomatic breast lesions by the experienced radiologists.

	**Radiologists diagnosis**	**Pathological result**	
		**Benign**	**Malignant**
Asymptomatic lesions	BI-RADS 3	103	4
	BI-RADS 4a	40	2
	BI-RADS 4b	9	9
	BI-RADS 4c	3	27
	BI-RADS 5	0	38
Symptomatic lesions	BI-RADS 3	46	4
	BI-RADS 4a	21	8
	BI-RADS 4b	6	22
	BI-RADS 4c	3	39
	BI-RADS 5	0	67

### Comparing the Performances of the Deep Learning-Based CAD System and Radiologists

#### For Asymptomatic Patients

The specificity (83.87 vs. 66.45%, *p* < 0.001), PPV (75.00 vs. 59.38%, *p* = 0.013) and accuracy (87.23 vs. 76.17%, *p* = 0.002), and AUC (0.89 to 0.81, *p* = 0.0013) of the CAD were all significantly higher than those of the radiologists. The sensitivity and NPV were not significantly different between the CAD and the radiologists (*p* > 0.05).

#### For Symptomatic Patients

The sensitivity (97.14 vs. 80.00%, *p* < 0.001), NPV (92.00 vs. 62.67%, *p* < 0.001) and accuracy (84.26 vs. 73.61%, *p* = 0.002), and AUC (0.79 to 0.71, *p* = 0.040) of the radiologists were all significantly higher than those of the CAD. The specificity and PPV were not significantly different between the radiologists and the CAD system (*p* > 0.05).

### Comparing the Performances of the Deep Learning-Based CAD System for Asymptomatic Patients and for Symptomatic Patients

The sensitivity (93.75 vs. 80.00%), specificity (83.87 vs. 61.84%), and accuracy (87.23 vs. 73.61%), and AUC (0.89 vs. 0.71) of the CAD were higher for asymptomatic patients than for symptomatic patients.

### For the Asymptomatic Screening Patients With Lesions <1 cm

In this study, a total of 87 lesions were <1 cm, of which, 61 were in asymptomatic patients. In the asymptomatic patients with lesions <1 cm, both the specificity (88.64 vs. 65.91%, *p* = 0.002) and accuracy (91.80 vs. 75.41%, *p* = 0.014) of the system were significantly higher than those of the experienced radiologists.

### For the BI-RADS 4a Lesions of the Asymptomatic Patients

For the asymptomatic patients, 42 lesions were diagnosed as BI-RADS 4a by the radiologists. The pathologic results showed that 40 lesions (95.23%) were benign and 2 lesions (4.76%) were malignant. If the BI-RADS 4a lesions diagnosed by the radiologists in asymptomatic patients were downgraded to BI-RADS 3 according to the CAD system results, then 54.8% (23/42) of the lesions would avoid biopsy without missing the 2 malignant tumors.

## Discussion

As an important supplementary modality for mammography, ultrasound has the advantages of avoiding radiation and being simple and easy to use. Performing bilateral whole breast screening for Asian women with small breasts is easy, and the breast lesions can be observed in detail (12). However, ultrasound requires extensive experience since this modality is an operator-dependent examination with lower reproducibility, specificity and PPV than mammography (13). In recent years, CAD has been used to overcome this shortcoming and to increase diagnostic accuracy (14, 15), similar to elastography, which has been used as an adjunct tool to decrease the number of unnecessary biopsies while improving the specificity of ultrasound without losing sensitivity (16). Shibusawa et al. reported that CAD could significantly increase the AUC of the observers from 0.649 to 0.783 (*p* = 0.0167) (12). A recent study showed that adding CAD results to ultrasound significantly improved the specificity, accuracy, and PPV of radiologists without losing sensitivity and NPV (17).

### The Role of Deep Learning-Based CAD System in the Breast Lesions

The emergence of deep learning methods has profoundly influenced the medical field. Currently, deep learning techniques are considered the most advanced technology for image classification (18). Deep learning-based CAD systems are different from conventional CAD systems based on manual feature design. Deep learning-based CAD is superior to conventional CAD (19). The deep learning-based CAD system used in our study (Samsung corporation, Seoul Korea) is a newly developed CAD system for breast ultrasound based on deep learning of raw ultrasound signals through a convolutional neural network. After extensive learning and training on a large number of databases, the deep learning-based CAD system could extract high-order statistics and optimize the balance of input and output data through multiple hidden layers to provide an accurate diagnosis (9). The original unprocessed ultrasound signals were collected as the raw data and information for the deep learning-based CAD system to analyze through a complex hierarchical framework. Therefore, the deep learning-based CAD system did not have interference from artifacts or man-made interference, which leads to more realistic and reliable diagnoses. The analysis process of the deep learning-based CAD system is different from how by radiologists makes observations with their naked eyes, and more inherent information can be obtained by the CAD system. The analyses and descriptions of deep learning-based CAD include shape, echo and texture features using spatial gray-level dependence matrices, intensity in the tumor area, gradient magnitude in the tumor area, orientation, distance between the tumor shape and a best-fit ellipse, average gray value changes or histogram changes between the tissue and tumor area, comparison of the gray values of the tumor surroundings, the number of lobulation/protuberances/depressions, and the lobulation index (20). Moreover, deep learning-based CAD is economical, easy-to-operate, and capable of providing a rapid diagnosis; thus, this method can be easily incorporated in clinical practice (8). Segni et al. (21) reported that deep learning-based CAD had good performance. The sensitivity, specificity, PPV, NPV and AUC were 91.1, 70.8, 85.4, 81.0, and 0.81%, respectively. The AUC was consistent with that found in our study (0.81).

Ultrasound screening has a low specificity and PPV (4–6). Previous studies have shown that deep learning-based CAD could improve the specificity of ultrasound. Kim et al. (22) reported that the specificity (65.8 vs. 30.9%), PPV (58.3 vs. 46.2%), accuracy (70.8 vs. 56.2%) and AUC (0.725 vs. 0.653) of the deep learning-based CAD system were all significantly higher than those of the experienced radiologists (*p* < 0.05) when using BI-RADS 4a as the cutoff value. This finding indicated that deep learning-based CAD had good clinical value. Cho et al. (8) also showed that the sensitivity, specificity, PPV, NPV, accuracy and AUC of deep learning-based CAD were 72.2, 90.8, 86.7, 79.7, 82.4, and 0.815%, respectively. The specificity, PPV, and accuracy of the deep learning-based CAD system were all significantly higher than those of 2 experienced radiologists (*p* < 0.05). Thus, deep learning-based CAD could increase the specificity, PPV, and accuracy of ultrasound. For the asymptomatic patients in our study, the sensitivity, specificity, PPV, NPV, accuracy and AUC of the deep learning-based CAD system were 93.8, 83.9, 75.0, 96.3, 87.2, and 0.89%, respectively. The specificity (83.9 vs. 66.5%, *p* < 0.001), PPV (75.0 vs. 59.4%, *p* = 0.013), accuracy (87.2 vs. 76.2%, *p* = 0.002) and AUC (0.89 vs. 0.81, *p* = 0.0013) of the deep learning-based CAD system were all significantly higher than those of the radiologists. In our study, in the asymptomatic patients, the PLR (5.81 vs. 2.83) and PPV (75.00 vs. 59.38) of CAD were higher than those of radiologists. This means that, in the asymptomatic patients, the probability of a malignant diagnosis of CAD to be a true malignant lesion is higher than that of radiologists. In the symptomatic patients, the NLR (0.05 vs. 0.32) was lower of radiologist than that of CAD and the NPV (92.00 vs. 62.67) of radiologists was higher than that of CAD. This means that, in the symptomatic patients, the probability of a benign diagnosis of radiologist to be a true benign lesion is higher than that of CAD.

### For Asymptomatic Patients

To the best of our knowledge, this is the first study to report the performance of a deep learning-based CAD system in the comparison of asymptomatic and symptomatic patients with breast lesions. Our study showed that the CAD system was more effective for asymptomatic patients than for symptomatic patients. Compared with those for the symptomatic patients, the sensitivity (93.8 vs. 80.0%), specificity (83.9 vs. 61.8%), accuracy (87.2 vs. 73.6%) and AUC (0.89 vs. 0.71) of the asymptomatic patients were all increased. These results indicate that the CAD system had a better performance in patients without clinical symptoms and medical or family histories. The CAD system is better than the human naked eye at extracting and analyzing inherent patterns from raw information data. Therefore, in the asymptomatic screening breast lesions, the diagnostic performance of radiologists could be improved by using a deep learning-based CAD approach.

### For Symptomatic Patients

To diagnose breast lesions, many clinical factors are taken into account in addition to the images, such as the patient's age, symptoms, surgical histories, family histories, high-risk factors, clinical examination results, and other imaging findings, including those from mammography, MRI, color Doppler ultrasound, and elastography. The diagnosis is a comprehensive analysis and judgment. In our study, there were 5 malignant phyllodes tumors, 4 of which were postoperative recurrence. All 4 solid tumors had regular shapes and clear boundaries on the images. The radiologists correctly diagnosed these lesions as recurrent malignant phyllodes tumors, while the CAD misdiagnosed these lesions as benign tumors. In this study, one patient who previously underwent modified radical mastectomy for breast cancer 4 years ago had recurrence on the chest wall. The recurrent tumor manifested as a solid nodule with a regular shape, clear boundary, and rich internal blood flow. The radiologists correctly diagnosed this mass as a recurrent cancer, while the CAD also misdiagnosed this mass as a benign tumor. There were 15 inflammatory lesions in the present study, of which 7 were misdiagnosed as malignant by the CAD. These 7 lesions had irregular shapes and ill-defined borders; these lesions tended to be misdiagnosed as breast cancer without any medical histories or clinical symptoms. These observations indicated that the clinical diagnostic process and CAD techniques were significantly different. The clinical diagnostic process strongly depends on the medical history and clinical manifestations. In contrast, the CAD system only analyses imaging features without considering any non-imaging factors. Thus, the CAD has a better performance in the asymptomatic screening breast lesions. Adding clinical information into the CAD diagnostic process may be helpful in the future.

### For the Asymptomatic Screening Patients With Lesions <1 cm

Small cancer with an invasive component <1 cm is considered unlikely to metastasize, and more than 90% of small cancers do not have axillary lymph node metastases, regardless of the histological grade (23). Therefore, detecting small cancers at the early stage is very important for the screening program. With the tumor size decreases, the characteristics of the cancer are also likely to decrease, such as desmoplastic changes and surrounding tissue changes to invasion (24). Therefore, correctly diagnosing small cancers is a true challenge for radiologists. In our study, the screening asymptomatic lesions were significantly smaller than the symptomatic lesions (1.44 vs. 2.42 cm, *p* < 0.05), which reveals the significance of breast screening for detecting small and early-stage breast cancer. In total, 87 lesions were smaller than 1 cm in our study, of which 61 lesions were from asymptomatic patients. Both the specificity (88.64 vs. 65.91%, *p* = 0.002) and accuracy (91.80 vs. 75.41%, *p* = 0.014) of the CAD were significantly higher than those of the experienced radiologists. These results suggest that for small breast cancers, the deep learning-based CAD system is more capable at extracting hidden information contain in the raw imaging data and recognizing the features of small cancers, which are indistinguishable to the radiologist's naked eye. The miniscule signs of malignant small breast cancer may be more easily identified by a deep learning-based CAD system than the naked human eye. Therefore, the diagnostic performance of radiologists for small cancer could be improved by a deep learning-based CAD system.

### For the BI-RADS 4a Lesions of the Asymptomatic Patients

BI-RADS 4a lesions are worrisome lesions, most of which are benign. Correct diagnoses of BI-RADS 4a lesions can reduce unnecessary biopsies and decrease the false-positive rate, which has always been the goal of radiologists. In the asymptomatic patients of this study, 95.23% (40 of 42) of the BI-RADS 4a lesions were benign. If the diagnosis process for BI-RADS 4a lesions also involved the CAD results, then 54.76% (23 of 42) of the benign lesions could avoid being unnecessarily biopsied without missing any malignant tumors. Thus, deep learning-based CAD is helpful in distinguishing benign from worrisome lesions. Choi et al. (17) also found that deep learning-based CAD could improve the diagnostic performance of leading radiologists and enable radiologists to correctly diagnose lesions that are difficult to classify as BI-RADS 3 or 4a.

There were several limitations in this study. First, the proportion of ductal carcinoma *in situ* in this study was slightly low (30/220), which may be because ultrasound is not well-suited for detecting ductal carcinoma *in situ*, whose main feature is microcalcification. The CAD did not perform well for detecting ductal carcinoma *in situ* (21/30). Therefore, the results of this study may overestimate the diagnostic efficacy of the CAD. Second, the image acquisition for the CAD is also operator dependent. In the present study, the representative images analyzed by CAD were selected by two experienced radiologists with more than 12 years experience in breast ultrasound. The representative image might be better in this study, and the diagnostic performance of the CAD needs further verification. Third, the number of cases is limited and the sample size needs to be expanded in future studies or multicenter studies.

In conclusion, a deep learning-based CAD system has the advantages of convenient operation and accurate diagnosis of breast lesions, especially in the asymptomatic screening patients. For asymptomatic patients, we could rely more on the CAD results in the future. For patients with medical histories or symptoms, we should make comprehensive judgments based on the clinical histories and symptoms. The deep learning-based CAD approach also has good diagnostic performance for small breast cancer (<1 cm). Therefore, a deep learning-based CAD system has good screening value for asymptomatic breast cancer at an early stage.

## Data Availability Statement

The raw data supporting the conclusions of this article will be made available by the authors, without undue reservation, to any qualified researcher.

## Ethics Statement

The studies involving human participants were reviewed and approved by the ethics committee of Peking Union Medical College hospital. The patients/participants provided their written informed consent to participate in this study.

## Author Contributions

MX and CZ: drafting the manuscript and organizing the database. JL and YJ: revising the work critically for important intellectual content. JZ: acquisition of data for the work. HL: interpretation of data for the work. MW, YO, and YZ: analysis and interpretation of data for the work. MX and QZ: acquisition, analysis, and interpretation of data for the work. QZ: substantial contributions to the conception or design of the work. All authors contributed to the article and approved the submitted version.

## Conflict of Interest

The authors declare that the research was conducted in the absence of any commercial or financial relationships that could be construed as a potential conflict of interest. The handling editor declared a shared committee affiliation, though no other collaboration, with one of the authors, QZ, at the time of review.

## Abbreviations

CAD: deep learning framework-based computer-aided diagnosis
SE: sensitivity
SP: specificity
PLR: positive likelihood ratio
NLR: negative likelihood ratio
95% CI: 95% confidence interval.
